# Invasive Fungal Infections During Extracorporeal Membrane Oxygenation: A Case Series from Intensive Care Unit and Literature Review

**DOI:** 10.3390/diagnostics16040505

**Published:** 2026-02-07

**Authors:** Francesca Serapide, Riccardo Serraino, Angelo Feola, Helen Linda Morrone, Vincenzo Olivadese, Giuseppe Neri, Eugenio Biamonte, Andrea Bruni, Eugenio Garofalo, Federico Longhini, Alessandro Russo

**Affiliations:** 1 Infectious and Tropical Disease Unit, Department of Medical and Surgical Sciences, “Magna Graecia” University of Catanzaro, 88100 Catanzaro, Italy; f.serapide@unicz.it (F.S.); r.serraino1@gmail.com (R.S.); angelo.feola@studenti.unicz.it (A.F.); helen.morrone@gmail.com (H.L.M.); olivadesevincenzo.95@gmail.com (V.O.); 2 Intesive Care Unit, Department of Medical and Surgical Sciences, “Magna Graecia” University of Catanzaro, 88100 Catanzaro, Italy; giuseppeneri91@gmail.com (G.N.); biamonte.eugenio@gmail.com (E.B.); andrea.bruni@unical.it (A.B.); eugenio.garofalo@unicz.it (E.G.); flonghini@unicz.it (F.L.); 3 Department of Pharmacy, Health and Nutritional Sciences, University of Calabria, 87036 Arcavacata di Rende, Italy

**Keywords:** ECMO, invasive fungal infections, candida, invasive aspergillosis, case series, respiratory infections

## Abstract

**Background**: Extracorporeal membrane oxygenation (ECMO) support is associated with potentially life-threatening complications, among which nosocomial infections play a significant role. The increasing incidence of fungi as causative agents of ECMO-associated infections is a growing concern. **Methods**: This case series includes all patients admitted to the Intensive Care Unit (ICU) of the “Renato Dulbecco” Teaching Hospital in Catanzaro who developed invasive fungal infections (IFIs) during ECMO support. **Results**: Of the 70 patients, 15.7% (*N* = 11) developed IFIs during ECMO. Among these, 91% (*N* = 10) died, while one patient survived and was discharged. Of the IFIs, 72.7% (*N* = 8) were cases of invasive candidiasis (IC), and 18.2% (*N* = 2) were cases of invasive pulmonary aspergillosis (IPA). One patient developed both IC and IPA during ECMO treatment. Additionally, 54.5% (*N* = 6) of the patients with IFIs also had bacterial co-infections, most of which were caused by multidrug-resistant (MDR) Gram-negative bacteria. **Conclusions:** This study highlights the high incidence and mortality of IFIs in ECMO patients. It underscores the urgent need for clear definitions, better diagnostic strategies, pharmacokinetic data on antifungal therapies, and the implementation of therapeutic drug monitoring (TDM) to optimize outcomes in this vulnerable population.

## 1. Introduction

Extracorporeal membrane oxygenation (ECMO) provides support for respiratory and/or cardiac function when the native organs are unable to perform adequately. Its use has increased in the late 2000s, particularly during the H1N1 influenza pandemic, where it proved effective in managing severe cases of acute respiratory distress syndrome (ARDS) [1,2].

ECMO support is associated with potentially life-threatening complications, among which nosocomial infections play a substantial role [3,4,5]. In addition to infections caused by Gram-negative bacteria, invasive fungal infections (IFIs) primarily due to *Candida* spp., are frequently observed [6,7]. The rising incidence of fungi as causative microorganisms of ECMO-associated infections is a significant concern. Managing these infections presents considerable challenges, not only due to the technical complexity of replacing ECMO’s mechanical components but also because of the limited data available on the pharmacokinetics and pharmacodynamics (PK/PD) of antifungal therapies in this setting. However, the prognostic significance of IFIs while undergoing ECMO is still unclear.

This case series provides a comprehensive description of all patients admitted to our hospital’s Intensive Care Unit (ICU) who developed an IFI while receiving ECMO support, highlighting certain aspects of therapeutic management that warrant further clinical investigation. The primary objective of this case series is to present a real-world experience of the management of these complications by detailing the clinical characteristics and treatment outcomes of patients and comparing them with data available in the existing literature.

## 2. Materials and Methods

This case series includes all patients admitted at “Renato Dulbecco” Teaching Hospital of Catanzaro, Italy, in the ICU who developed IFIs during ECMO support. A retrospective analysis of IFI cases was conducted from October 2020 to March 2024 in adult patients (over 18 years of age) who received Veno-Venous ECMO (V-V ECMO) for respiratory support during the study period. Data were extracted from the clinical charts of patients who fit in the description above. Informed consent was obtained from the patient (or relative/guardian) for the publication of all images, clinical data and other data included in the main manuscript.

All ECMOs were placed exclusively in ICU, and a femoro-jugular configuration was preferred. The ECMO circuit was kept isolated as much as possible. In no case was perioperative antimicrobial prophylaxis administered at the time of cannulation. Routine microbiological monitoring was uniformly conducted. All patients with clinical suspicion of pneumonia underwent weekly bronchoscopy with fluid collection from bronchoalveolar lavage (BAL) on which microbiological tests, including galactomannan (GMN) antigen and culture tests, were performed. Serum GMN and serum Beta-D-glucan (BDG) detection were also performed with same timeline. Detection of GMN on BAL and on serum was performed with immunoenzymatic test (BAL: ≥1.0 index; serum ≥ 0.50 index). Moreover, in patients with suspected invasive fungal infection, fungal blood cultures were performed. Finally, in cases of suspected bacterial co-or superinfection, blood cultures and/or bronchoalveolar lavage (BAL) cultures were obtained. Patients were re-evaluated by an Infectious Diseases consultant team every 24–48 h and the microbiological tests were repeated based on patients’ clinical course.

We included all IFIs that occurred between the timeframe from 24 h after cannulation to 24 h before decannulation. The diagnoses of invasive candidiasis (IC) and invasive pulmonary aspergillosis (IPA) were formulated in agreement with the 2024 consensus definitions of invasive fungal diseases in adult patients in ICU [8].

## 3. Results

**Figure 1:**
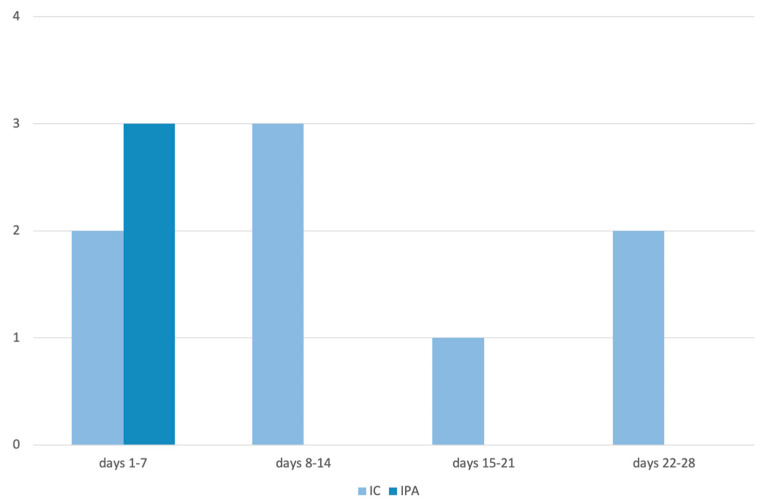
Time of onset in days of IFI from the start of ECMO. **Legend**. IC: invasive candidiasis; IPA: invasive pulmonary aspergillosis. This figure shows the time of onset from the start of ECMO for IC and IPA. All IPA (3) occurred in the first week, whereas IC occurred mainly from the second week onwards.

**Figure 2:**
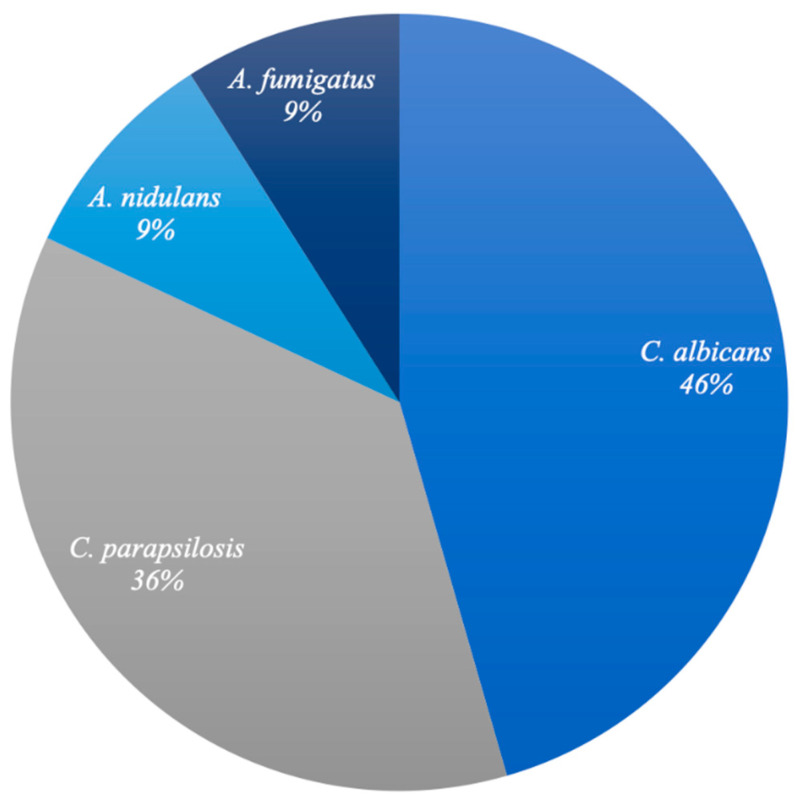
Etiologies of IFIs detected. This figure shows the etiologies of the IFIs found. For CI, 46% was due to *C. albicans* and 36% to *C. parapsilosis*. For IPA, 9% was due to *A. fumigatus* and 9% to *A. nidulans*.

Characteristics of patients with IFI in ECMO are reported in Table 1, Table 2 and Table 3. From October 2020 to March 2024, 70 patients received V-V ECMO treatment in the ICU of the “*Renato Dulbecco*” Teaching Hospital in Catanzaro; no cases requiring artero-venous ECMO was registered during the study period. Of the 70 patients, 15.7% (*N* = 11) developed IFIs during ECMO. A total of 72% of patients who developed fungal infection were male with a mean age of 57.2 years (SD 12.3). Only one patient was discharged whereas the other 10 died (91%). Among IFIs, 72.7% (*N* = 8) were ICs, 18.2% (*N* = 2) were probable IPAs and 9.1% (*N* = 1) presented with both IC and probable IPA during ECMO treatment. The mean time to onset of IC from the start of ECMO treatment was 12.6 days (Figure 1). The etiological agents found are depicted in Figure 2.

All patients (*N* = 9) with candidemia had a prior isolation of the same *Candida* species from a different district: six of the above exhibited a respiratory tract colonization (*Candida* spp. isolate on BAL culture), two patients had a prior isolation of *Candida* in a urine sample, and one patient had both respiratory tract and urinary tract colonization.

Finally, 54.5% (*N* = 6) of patients who developed IFI during ECMO also had bacterial co-infections. Most of these were due to multidrug-resistant Gram-negative bacteria (Table 1).

Here, we report three emblematic cases observed in our ICU in the study period that highlight the challenges in the therapeutic management of this disease, especially in ICU patients.

### 3.1. Case Series Presentation

#### 3.1.1. Case 4 in Table 1

A 58-year-old male with no prior medical history was admitted to the ICU for acute respiratory failure due to COVID-19 pneumonia. On day 1 (D1), due to clinical worsening, the patient required orotracheal intubation (OTI) and was placed on V-V ECMO. Chest X-ray revealed diffuse interstitial thickening consistent with severe viral pneumonia. On day 13 (D13), blood cultures tested positive for carbapenem-resistant *Acinetobacter baumannii* and *Enterococcus faecium*; thus, targeted antibiotic therapy with cefiderocol, tigecycline, and vancomycin was initiated. On day 22 (D22), new blood cultures were obtained and tested positive for *Candida albicans*, along with a positive serum BDG assay. Antifungal therapy with caspofungin at standard dosage was started. However, blood cultures remained persistently positive for *C. albicans*. Despite intensive supportive care and antimicrobial therapy, the patient’s condition continued to deteriorate, and he expired during the fourth week of hospitalization.

#### 3.1.2. Case 2 in Table 2

A 65-year-old male with a history of chronic ischemic heart disease and previous severe COVID-19 pneumonia requiring ICU admission was initially hospitalized in a different center for acute respiratory failure due to H1N1 influenza pneumonia detected by molecular testing. Antiviral therapy with oseltamivir was initiated. Due to worsening respiratory function, the patient underwent OTI on day 4 (D4) and was transferred to our center on day 6 (D6) for initiation of V-V ECMO. On day 8 (D8), bronchoscopy with bronchoalveolar lavage (BAL) was performed, revealing positivity for galactomannan (GM) antigen, despite negative fungal cultures. Given the compatible radiological findings (multiple areas of ground glass opacity and consolidation of varying sizes are visible in both lungs, particularly in the right basal region, affecting most of the posterior segment. A large area of consolidated/atelectatic parenchyma with signs of air bronchogram is visible, associated with abundant ipsilateral pleural effusion. No pleural effusion on the left. Diffuse interstitial thickening bilaterally) and clinical suspicion of invasive pulmonary aspergillosis (IPA), antifungal therapy with isavuconazole at standard dosage was started. ECMO was discontinued on day 15 (D15) following clinical improvement. After a prolonged ICU stay, the patient was transferred to another hospital facility in the third month of hospitalization.

#### 3.1.3. Case 1 in Table 3

A 58-year-old male underwent emergency cardiac surgery on day 1 (D1) for acute aortic dissection. On day 3 (D3), he required intubation and V-V ECMO due to severe respiratory failure and continuous renal replacement therapy (CRRT) due to anuria. Empiric antibiotic therapy was started for suspected ventilator-associated pneumonia. On day 6 (D6), a positive GMN antigen was detected on BAL sample and cultures subsequently showed growth of *Aspergillus fumigatus*, prompting the initiation of antifungal therapy with isavuconazole at standard dosage. On day 8 (D8), blood cultures were positive for *Candida albicans*, and liposomal amphotericin B was started. BDG assay was also positive. Despite intensive antimicrobial and organ support therapy, the patient expired during the second week of hospitalization.

## 4. Discussion

In this case series, we observed a high incidence of IFIs (total 11/70, 15.7%—IC 9/70, 12.8%—IPA 3/70, 4.3%). Mortality among our IFI-ECMO patients was also strikingly high, reaching 91% (10 out of 11 patients) despite early diagnosis and timely initiation of targeted antifungal therapy.

In a 2024 retrospective analysis of 392 patients who received V-A ECMO the IFI incidence was 4.6% (IC and IPA 3.8% 1.0% respectively), the most frequent etiological agent being *C. albicans*. In this study, severe infection onset, moderate-to-severe renal impairment and CRRT were predictive of IFI, and the mortality rate was extremely high (94%) [9].

In a recent retrospective analysis including 250 patients who underwent ECMO, an incidence of 5.7% of candidemia (mainly due to *C. albicans*) was found in patients on V-A ECMO and 16% (mainly due to *C. glabrata*) in patients undergoing V-V ECMO. In another similar study, candidemia had an incidence of 5.4%, with no major differences in species between V-V ECMO and V-A ECMO. In both studies, a prolonged course of V-V ECMO was associated with a higher incidence. The mortality rate was approximately 70% in both studies [4,10].

In a 2023 retrospective cohort study of COVID-19 patients, a high incidence of candidemia (36%) was observed in patients who underwent V-V ECMO, compared to patients who did not require this support, a difference that could be attributed to the altered immune system observed in these patients; furthermore, *C. parapsilosis* was the most frequent etiological agent [11]. In a retrospective study from the same year that included 452 patients undergoing ECMO, the incidence of IFIs was 4.8% and 11.0% in patients without and with COVID-19 pneumonia, respectively. In patients with COVID-19, 84.6% of the IFIs were ICs and 15.4% were IPAs. The study showed that COVID-19 status was an independent risk factor for IFI (OR 4.30; *p* = 0.002). The mortality rate was 100% [12].

In the 2018 ELSO registry analysis, no increased incidence of invasive aspergillosis or IC was found in patients on ECMO compared to the general ICU population. Incidence was 0.4% for IPA and 1.2% for IC. Risk factors for *Aspergillus* infection included solid organ transplant, respiratory support, and influenza infection. Risk factors for candidemia included sepsis and CRRT. In the multivariate analysis, *Aspergillus* involvement (OR 0.40; *p* < 0.001) and candidemia (OR 0.47; *p* < 0.001) were both independently associated with decreased survival [13].

A 2022 single-center retrospective study of 145 patients receiving V-V ECMO showed a 9% incidence of candidemia (13 patients of whom 4 developed infections within 5 days of decannulation). Infections were due mostly to *C. albicans* and *C. parapsilosis* (38% and 38%). This study showed a weekly increased incidence throughout the ECMO duration, with an increasing trend in OR (*p* = 0.005), and a window of prolonged risk after decannulation [14].

Moreover, as shown in Table 1, two patients with documented candidemia had negative BDG results. This finding is consistent with the existing literature, particularly in non-abdominal-source infections [15]. In our case, the hypothesis could be a combination of several factors rather than a single factor such as lower microbial load (e.g., patients with a single positive blood culture), non-deep infections, lower clinical severity and the use of different BDG threshold values for different types of invasive Candida infections.

In summary, in the studies reviewed the incidence of IC ranges from 1.2% to 36% with some difference between V-A and V-V ECMO: in patients undergoing V-V ECMO the incidence of IC would appear to be higher; the incidence of IPA ranges from 0.4% to 1%. These data are in line with what was reported in our case series. In addition, incidence of IFIs could probably be underestimated as these were retrospective studies where sample collection was performed at the discretion of the treating physician. Furthermore, there is no clear definition of ECMO-related infection in the literature; some authors refer to ECMO-related or ECMO-associated nosocomial infections as any nosocomial infection occurring during the ECMO support period. In other cases, infection in patients undergoing ECMO is inconsistently defined within windows from 12 to 48 h after cannulation, and until 24 h to 30 days after ECMO withdrawal [16]. This contributes to not having a clear epidemiology of these infections. In our case series we considered infections arising within 24 h of cannulation until 24 h before decannulation.

In the published literature, renal failure and CRRT as well as COVID-19 seem to be associated with a higher incidence of IFI during ECMO [12,13]. In addition, a prolonged course of V-V ECMO also appears to account for a higher incidence. Moreover, in our case series a higher occurrence of IC was seen in patients after at least 10 days on ECMO.

Finally, in our study all patients with candidemia had a prior colonization by the same *Candida* species in at least one other site (BAL or urine). Data in the literature indeed suggest that *Candida* colonization is strongly associated with the likelihood of IC among ICU patients [17].

Therefore, close monitoring of patients is necessary in view of the high mortality shown in most studies, especially in patients with crucial risk factors. However, the ELSO currently advises against surveillance blood cultures, stating that there is a lack of evidence for their use or that the evidence suggests a lack of benefit [18].

In our case series, the main drugs used were caspofungin at usual dosage for candidemia (70 mg on day 1, then 50 mg once daily) and isavuconazole at the usual dosage for IPA (loading dose of 200 mg three times daily for six doses, followed by 200 mg once daily, 12–24 h after the last loading dose), and in some more complex cases, liposomal amphotericin B (L-AmB) at dosage of 5 mg/kg/day was used. Therapeutic drug monitoring (TDM) was not performed in the cases analyzed.

Data in the literature regarding the pharmacodynamics of antifungals in patients under ECMO treatment and the impact ECMO has on the PD of these drugs are very scarce.

Regarding L-AmB there are only a few case reports in the literature that present conflicting conclusions [19,20,21]. One of these shows that ECMO did not seem to have modified the PK of L-AmB suggesting that no dose adjustment should be necessary in those cases [18]. Moreover, a clinical case reported the occurrence of an ECMO circuit failure likely caused by filter clogging in a patient treated with L-AmB [21].

Another option could be the deoxycholate formulation, which is also highly protein-bound (more than 95%), but is more hydrophilic. According to some studies, it would appear to be preferable to liposomal formulation in terms of pharmacokinetics.

Among azoles, voriconazole, a second-generation triazole, showed significant sequestration in ECMO circuits due to its lipophilicity and high protein binding. Ex vivo studies reported up to 71% drug loss [22,23,24], and other studies often show subtherapeutic levels [25,26], necessitating dose adjustments. Factors like binding site saturation and membrane changes may influence drug levels, although some studies report no significant PK alterations during ECMO [26].

Posaconazole, a highly lipophilic and protein-bound triazole, showed substantial drug loss (64%) in ex vivo ECMO circuit studies [27]. However, a recent clinical study did not observe significant PK changes in ECMO patients compared to non-critically ill individuals. Still, some patients failed to achieve target plasma levels after the loading dose [28].

Fluconazole, the least lipophilic and protein-bound azole, showed minimal sequestration in ECMO circuits according to ex vivo studies, with drug recovery rates above 90% [22,29]. While adult data are limited [30,31], pediatric studies indicated that ECMO increases fluconazole’s volume of distribution (Vd), likely due to hemodilution, without significantly altering clearance [32].

Isavuconazole is a highly lipophilic and protein-bound triazole. While some case suggested drug sequestration in ECMO circuits leading to subtherapeutic levels [33,34], a recent observational prospective study in critically ill adults found no significant differences in drug concentrations across the ECMO membrane, with adequate trough levels achieved early [35].

Among the echinocandins, caspofungin, a hydrophilic antifungal with high protein binding, has shown conflicting pharmacokinetic data in the ECMO setting. Ex vivo studies report significant drug loss (up to 80%) [27], suggesting sequestration, while other studies show stable levels [23]. The old clinical reports available also vary, with some noting subtherapeutic concentrations and others achieving adequate exposure with standard dosing [36,37,38]. A recent prospective study found no significant PK differences in ECMO patients [39].

Some authors recommend increased dosages of antifungal agents for the treatment of infections during ECMO, e.g., a recent literature review suggested a caspofungin dosage of 70 mg (<80 kg of body weight) to 100 mg (≥80 kg of body weight) daily after a loading dose of 100 mg and 400 mg isavuconazole as the first dose followed by 200 mg q8 h for the next 5 doses and by a maintenance dose of 200 mg q24h) [40].

In summary, as a recent systematic review concluded, data on PK of antifungals during ECMO treatment are scarce and hold a low level of evidence, which is why TDM is a key weapon to avoid underdosing in these critical patients [41]. However, no data are available in the literature concerning the frequency of TDM sampling for each antifungal agent in patients on ECMO. Furthermore, the use of TDM worldwide is still limited to a small number of drugs and hampered by economic inequality in physicians’ access to MIC values and timely drug assay results [42]. It is also important to emphasize that subtherapeutic use of antimycotics carries a risk for the patient but also for the community as this leads to the emergence of resistance in pathogenic fungi [43].

The study has several limitations: the patient cohort is small and involves a single center; the retrospective nature of the study did not allow for the collection of complete patient data; no information is available on the total duration of ECMO for these patients or on a comparative control group; the selection of patients who are nevertheless very seriously ill and therefore have a higher intrinsic mortality rate related to their condition.

Despite the elevated overall mortality rate in this series of cases, it was not possible to assess the underlying cause of death for all cases and confirm that the mortality was attributable to a fungal infection.

This has only led to a simple descriptive analysis of the cases and, therefore, we are unable to comment on the relationship between specific clinical variables (such as COVID-19, CRRT, etc.) and the incidence of IFI.

## 5. Conclusions

Our case series highlights the critical issues related to IFIs in patients undergoing ECMO. Although the specific contribution of IFIs to patient prognosis remains only partially understood, the increasing utilization of ECMO and the rising incidence of fungal infections necessitate targeted interventions in several key areas, as summarized below:

- Establish a clear and precise definition of ECMO-related infection.
- Investigate whether, and to what extent, IFIs contribute to mortality in patients receiving ECMO.
- Clarify the impact of ECMO on the PK/PD of antifungal agents through ex vivo studies, to inform dosing recommendations for patients with IFIs on ECMO, and subsequently through clinical trials that also account for other conditions known to alter antifungal PK/PD (such as obesity, CRRT, etc.).
- Promote the implementation of TDM and provide data on its correct application.
- Assess, through targeted studies, preventive, and prophylactic strategies for non-neutropenic patients on ECMO who present specific risk factors for IFIs (such as prolonged V-V ECMO courses, multisite colonization, or the requirement for CRRT).

## Figures and Tables

**Table 1:** Invasive candidiasis: patients’ characteristics, laboratory findings and outcome.

No	Sex	Age	Medical History	ECMO Indication	Serum BDG	Blood Culture	Time of Infection Onset from ECMO (Days)	Bacterial Co-Infection	Antifungal Treatment	Outcome
**1**	M	65	NHL	Respiratory failure in COVID-19	negative	*C. albicans*	9	MSSA	Caspofungin (70 mg load, then 50 mg q24h)	Dead
**2**	M	45	No	Respiratory failure in COVID-19	positive	*C. parapsilosis*	9	*A. baumanni* *K. pneumoniae*	Caspofungin (70 mg load, then 50 mg q24h) Liposomal amphotericin B *	Dead
**3**	M	59	No	Respiratory failure in COVID-19	negative	*C. albicans*	4	No	Caspofungin (70 mg load, then 50 mg q24h)	Dead
**4**	M	58	No	Respiratory failure in COVID-19	positive	*C. albicans*	22	*A. baumannii* *E. faecalis*	Caspofungin (70 mg load, then 50 mg q24h)	Dead
**5**	F	72	HL, CKI	Respiratory failure in COVID-19	positive	*C. albicans*	8	*A. baumannii* *S. epidermidis*	Caspofungin (70 mg load, then 50 mg q24h)	Dead
**6**	F	48	No	Respiratory failure in COVID-19	positive	*C. parapsilosis*	21	No	Caspofungin (70 mg load, then 50 mg q24h) Fluconazole (800 mg load, then 400 q24h)	Dead
**7**	F	53	No	Respiratory failure in COVID-19	positive	*C. parapsilosis*	22	*K. pneumoniae* *A. baumannii*	Liposomal amphotericin B	Dead
**8**	M	30	Charcot-Marie-Tooth disease	Respiratory failure	positive	*C. parapsilosis*	6	*K. pneumoniae*	Caspofungin (70 mg load, then 70 mg q24h)	Dead

**Legend**. ECMO: extracorporeal membrane oxygenation; NHL: non-Hodgkin lymphoma; HL: Hodgkin lymphoma; BDG: beta-D-glucan; MSSA: methicillin-sensitive *S. aureus.* * therapeutic switch for persistent candidemia.

**Table 2:** Invasive pulmonary aspergillosis: patients’ characteristics, laboratory findings and outcome.

No	Sex	Age	Relevant Medical History	ECMO Indication	GMN (Serum or BAL)	BAL Culture	Time of Infection Onset from ECMO (Days)	Bacterial Co-Infection	Antifungal Treatment	Outcome
**1**	M	76	No	Respiratory failure in COVID-19	Positive (BAL)	*A. nidulans*	1	No	Isavuconazole 200 mg 3 times daily (six doses) load, then 200 mg q24h	Dead
**2**	M	65	Severe cardiovascular disease	Respiratory failure in Influenza virus H1N1 pneumonia	Positive (BAL)	No	1	No	Isavuconazole 200 mg 3 times daily (six doses) load, then 200 mg q24h	Discharged

**Legend**. ECMO: extracorporeal membrane oxygenation; BAL: bronchoalveolar lavage; GMN: galactomannan.

**Table 3:** Mixed infection: patients’ characteristics, laboratory findings and outcome.

*N*	Sex	Age	Relevant Medical History	ECMO Indication	GMN (BAL) or BDG (Serum)	Blood Culture BAL Culture	Time of First Infection Onset from ECMO, Days (IPA—IC)	Bacterial Co-Infection	Antifungal Treatment	Outcome
1	M	59	Severe cardiovascular disease	Respiratory failure	Positive (BDG)	*C. albicans* *A. fumigatus*	5 (IPA)	No	Isavuconazole 200 mg 3 times daily (six doses) load, then 200 mg q24h Liposomial amphotericin B	Dead

**Legend**. ECMO: extracorporeal membrane oxygenation; BAL: bronchoalveolar lavage; GMN: galactomannan; BDG: beta-D-glucan; IPA: invasive pulmonary aspergillosis; IC: invasive candidiasis.

## Data Availability

The data presented in this study are available on request from the corresponding author due to privacy reason.

## Abbreviations

The following abbreviations are used in this manuscript:

ECMO	Extracorporeal membrane oxygenation
ICU	Intensive Care Unit
IC	Invasive Candidiasis
IPA	Invasive Pulmonary Aspergillosis
IFI	Invasive Fungal Infection
TDM	Therapeutic Drug Monitoring
ARDS	Acute Respiratory Distress Syndrome
PK/PD	Pharmacokinetics/Pharmacodinamics
BDG	Beta-D-glucan
